# Cross-modal and intra-modal commutativity of magnitude productions

**DOI:** 10.3758/s13414-026-03243-6

**Published:** 2026-04-17

**Authors:** Wolfgang Ellermeier, Florian Kattner

**Affiliations:** 1 Institut für Psychologie, Technical University of Darmstadt, Alexanderstr. 10, 64283 Darmstadt, Germany; 2 Institute for Mind, Brain and Behavior, Health and Medical University, Potsdam, Germany

**Keywords:** Magnitude estimation, Cross-modality matching, Cross-modal commutativity, Intra-modal commutativity, Reference intensity, Axiomatic measurement, Psychophysics

## Abstract

Luce, Steingrimsson, and Narens (*Psychological Review, 117,* 1247–1258, 2010) postulated that if ratio magnitude productions involving two perceptual dimensions exhibit “cross-dimensional commutativity,” they may be represented on a single internal scale of subjective intensity. Commutativity here refers to the order independence of successive magnitude productions (e.g., adjusting the subjective intensity of a stimulus successively by factors of 2 and 3 should produce the same result regardless of which factor comes first [×2×3 = ×3×2]). In the present experiment, these operations were performed (1) within the same modality (here: loudness or brightness), and (2) across modalities—that is, making productions from light to sound (e.g., “make the sound twice as loud as the light is bright”) and back, or vice versa. In individual, within-subjects experiments involving repeated loudness and brightness productions, 13 participants made adjustments to evaluate both kinds of commutativity. In line with previous findings (Ellermeier, Kattner, & Raum, *Attention, Perception, & Psychophysics, 83*[7], 2955–2967, 2021), both intra-modal and cross-modal commutativity held for most participants, but the final results of corresponding sequences of cross-modal and intra-modal adjustments (e.g., of the type ×2×3) typically did not coincide. That inconsistency is interpreted as participants choosing different internal reference points when making cross-modal versus intra-modal magnitude productions, but it does not preclude their using a common internal yardstick. The aggregated and raw data of all participants are available in an OSF repository (https://osf.io/5avbw/?view_only=687aef266f174648863f86b4982e35b9). The study has not been preregistered.

In the past 3 decades, the interest in Stevens’s concept of suprathreshold scaling or magnitude scaling (Stevens, 1956, 1975) has been rekindled by theoretical proposals put forward by mathematical psychologists that promised to put Stevens’s largely implicit, and often untested, assumptions on a solid “axiomatic” fundament (e.g., Luce, 2002, 2004; Narens, 1996; Steingrimsson & Luce, 2007). From the formal theory, they derived certain empirically testable assumptions that must hold for Stevens’s magnitude scaling to work as postulated. If these axioms are shown to hold, it may be concluded that observers’ judgments are based on an internal ratio scale of sensation magnitude.

The publication starting this line of research was Narens’s (1996) theoretical work on magnitude estimation. Narens focused on axiomatizing a psychophysical task called “magnitude production” (Reynolds & Stevens, 1960; Stevens & Greenbaum, 1966; Teghtsoonian & Teghtsoonian, 1978; ASA Standard 5.34), but emphasized that the theory is meant to be applicable to other methods of magnitude scaling as well, such as magnitude estimation or cross-modality matching. In magnitude production, observers are asked to adjust a stimulus so that it elicits a numerically specified sensation, or—more commonly—so that it produces a certain ratio of sensation strengths relative to that evoked by a standard stimulus. In Stevens’s (e.g., 1975) conceptualization—formulated by Narens (1996) as “Stevens” assumptions—it is presumed (1) that if observers are instructed to produce (or estimate) sensation ratios, then they will operate on a ratio scale (Stevens, 1946) and (2) that the number words (numerals) used in presenting a magnitude production task may be treated as if they were true mathematical entities (numbers).

## Commutativity of magnitude productions

Narens’s (1996) influential conceptualization reformulated these implicit assumptions in a stringent “axiomatic” framework and showed that direct scaling procedures such as magnitude estimation or production are valid only if—along with a number of “technical” axioms—a behavioral and testable condition termed “commutativity” holds.

Commutativity (Narens’s Axiom 4) means that, given *x* is a stimulus level produced in a magnitude production trial and *p* and *q* are positive numbers, adjusting a physical stimulus to have *p* times the subjective magnitude as some standard intensity (thereby resulting in *x*_*p*_), and subsequently, starting from that outcome, to produce another stimulus level *q* times as strong subjectively (i.e., *x*_*p,q*_) should result in the same stimulus intensity as performing the two operations in the reverse order—as stated in Equation 1:

1 $${x}_{p,q} \sim {x}_{q,p}$$

For the case of magnitude productions of loudness that means that first doubling the loudness of a given standard, and on some subsequent trial tripling the outcome of that first trial should result in the same sound pressure level as initially tripling, and then doubling loudness starting from the same standard level.

Narens (1996) has shown that, if commutativity holds, subjects are operating on a ratio scale of sensation, an “inner psychological structure . . . for measuring the intensity of sensations” (Narens’s Axiom 7, p. 115); with the ratio property not being defined in an intuitive/operational sense as by Stevens (e.g., 1975) but formally as by axiomatic measurement theory (Narens, 1981; Narens & Luce, 1986). Thus, investigating commutativity for a given psychophysical task is addressing the first of Stevens’s assumptions. Testing “multiplicativity” (Narens’s Axiom 9; e.g., determining whether making a standard stimulus six times as intense is indistinguishable from first making it twice, then three times as intense) puts Stevens’s second implicit assumption to a test.

Empirical tests of Narens’s (1996) theory performed within a single perceptual dimension typically found commutativity to hold for most observers, while multiplicativity was often violated. This pattern of results was initially found for loudness (Ellermeier & Faulhammer, 2000; Steingrimsson & Luce, 2005, 2007; Zimmer, 2005), and later confirmed for brightness (Peißner, 1999; Steingrimsson, 2009, 2011; Steingrimsson et al., 2012), the visual size of circles (Augustin & Maier, 2008), the perceived duration of pure tones (Birkenbusch & Ellermeier, 2016; Birkenbusch et al., 2015), and for pitch intervals (Kattner & Ellermeier, 2014). According to Narens’s theory the fact that commutativity holds proves that observers are operating on an internal ratio scale, while the fact that multiplicativity is violated implies that the “numerals” that are used in magnitude estimation or production (the quantities of the “external” scale) may not be interpreted as the mathematical numbers they denote.

## Cross-dimensional commutativity

Luce et al. (2010) extended Narens’s (1996) theory of magnitude estimation and Luce’s (2002, 2004) “global psychophysical theory” to investigate—both theoretically and empirically—what they called “cross-dimensional” commutativity. It is meant to account for magnitude productions from one dimension into another and back, and is formally stated as follows:

2 $${x}_{p,q}^{fgf} \sim {x}_{q,p}^{fgf},$$

where the subscripts *q*, *p* refer to magnitude production factors as before, and the superscripts *f*, *g* represent the dimensions involved. In Luce et al. (2010), the *dimensions* refer to pure tones of different frequencies (e.g., 1 kHz vs. 2 kHz), the levels of which are being adjusted for magnitude productions of loudness. Note that in this application, while different physical stimulus dimensions (here: pure-tone frequencies) are involved, observers are to monitor their sensations on a single perceptual continuum, loudness. Thus, a “basic” cross-dimensional production trial (f→g) is formalized as ${x}_{p}^{fg}$ with, for example, *f* representing 1-kHz tones, *g* representing 2-kHz tones, and *p* being the production factor, say *p* = 3 (for making the 2-kHz tone 3 times as loud as the 1-kHz tone). Consequently, ${x}_{p,q}^{fgf}$ might be a mapping from one frequency to another and back ($f\to g\to f$), with *x* (on both sides of Eq. 2) being a sound pressure level. Likewise, an analogous case exists where the test of commutativity originates in the other dimension ($g\to f\to g$):

3 $${x}_{p,q}^{gfg} \sim {x}_{q,p}^{gfg}.$$

In addition, Luce et al. (2010) discuss a direct comparison of the cross-dimensional with the unidimensional case (Eq. 1), offering yet another opportunity to test the validity of cross-dimensional commutativity.

## Earlier tests of cross-dimensional commutativity

Luce et al. (2010) tested selected cases of their theory on a relatively small number of participants. As to the critical case of “cross-dimensional commutativity” (Equations 2 and 3), they found all four participants studied in that condition (two using multiples 2 and 3, and two using proportions 50% and 75%; see their Table B.2) to exhibit cross-dimensional commutativity. However, the cross-dimensional productions $f\to g\to f$ (Eq. 2) did not coincide with unimodal productions $f\to f\to f$ (Eq. 1). Luce et al. (2010) interpreted this result as indicating that sensation magnitudes on the two dimensions are measured on a common scale, but with different *reference points* when mapping a sensation from *f* into *g* and vice versa, an issue that will be taken up again.

When Steingrimsson et al. (2012) replicated these cross-dimensional tests using luminous squares of different hues presented on a computer monitor, the four participants for whom productions of the type $f\to g\to f$ were evaluated all exhibited cross-dimensional commutativity (as may be read off their Fig. 7), but the net result of these productions did not agree with the unimodal case either.

## Cross-modal commutativity

Ellermeier et al. (2021) extended the investigation to the kind of commutativity that is truly cross-modal, in that participants had to make magnitude productions of the type “Make the sound twice as loud as the light is bright!”, thereby involving two different sensory modalities, in this case, hearing and vision. This case is explicitly included in the modeling by Luce et al. (2010): Essentially, looking at cross-modal commutativity just means interpreting *f* and *g* in Equations 2 and 3 as two *different* sensory modalities in which observers map magnitude productions from one physical quantity onto the other, each having its own dimensionality and unit (e.g., sound pressure level [dB SPL] or luminance [cd/m^2^]).

Having 20 participants produce subjective multiples (e.g., 2×, 3×) of the loudness of noise bursts with respect to a standard brightness of a luminous square displayed on a computer screen, and vice versa, Ellermeier et al. (2021) performed individual tests of cross-modal commutativity by checking whether a sequence of 2×3× adjustments produced essentially the same final outcome as a sequence of 3×2× adjustments. They concluded that the evidence showed commutativity to hold by and large, since only seven of 40 (nonparametric) tests performed on 20 participants indicated statistically significant violations of commutativity. Parallel Bayesian analyses of each individual subject’s data (Ellermeier et al., 2022) also favored the null hypothesis of the commutativity axiom (Equations 2 and 3) to hold. The remaining violations of commutativity were tentatively attributed to participants’ inconsistencies in setting internal “reference points” for the different test conditions, a concept that had already been invoked by Luce et al. (2010).

## The concept of reference points

Recently, Heller (2021, 2025) showed that this analysis is incomplete, suggesting that four types of internal references should be distinguished in cross-modal situation like the one studied here: References might depend on which continuum they are established on, say loudness or brightness in the present investigation, and on whether they are located on the standard or variable dimension for a given magnitude production trial, the latter being their “role (in)dependence” in Heller’s (2021, 2025) terminology. To illustrate how references might operate in cross-modal magnitude productions, let us consider the instruction “Make the sound 3 times as loud as the light is bright!” Implicitly, that requires determining how bright the (standard) light intensity appears (“as the light is bright”) relative to some zero (or unity) reference: ${\rho }^{b\to l}$, with the arrow in the superscript indicating that the production is from brightness (the standard) to loudness (the variable stimulus), and the first variable (*b*) in the superscript indicating we are dealing with a reference on the brightness continuum. Furthermore, “Make the sound 3 times as loud” implies using a reference on the loudness continuum in relation to which “×3” is defined: ${\rho }^{l\leftarrow b}$. By the same token, when generating cross-modal magnitude productions from loudness to brightness, two further references are involved: ${\rho }^{l\to b}$ on the loudness and ${\rho }^{b\leftarrow l}$ on the brightness continuum.

If—for a given observer—these internal references are not ‘role-independent’ (i.e., ${\rho }^{b\to l}$ ≠ ${\rho }^{b\leftarrow l}$ or ${\rho }^{l\to b}\ne {\rho }^{l\leftarrow b}$), violations of commutativity will occur, Heller’s (2021) theory states. While this may sound like a post hoc explanation, it has recently been put to an empirical test: In a thorough reanalysis of the data collected by Ellermeier et al. (2021), Heller (2025; Table 3) estimated parameters for the unobservable reference intensities ρ, and found role dependencies for all but one participant, in that the internal reference intensity on a given continuum was consistently greater when it constituted the (variable) comparison than when it was the standard (e.g., ${\rho }^{b\leftarrow l}$> ${\rho }^{b\to l}$). Observing these violations of “cross-dimensional role independence” (Heller, 2021, Proposition 2) in the cross-modal magnitude productions and the somewhat inconclusive evidence in favor of commutativity led Heller (2025) to argue for a “near miss to cross-modal commutativity” in the Ellermeier et al. (2021) data.

## The missing comparison with intra-modal magnitude production

**Fig. 1:**
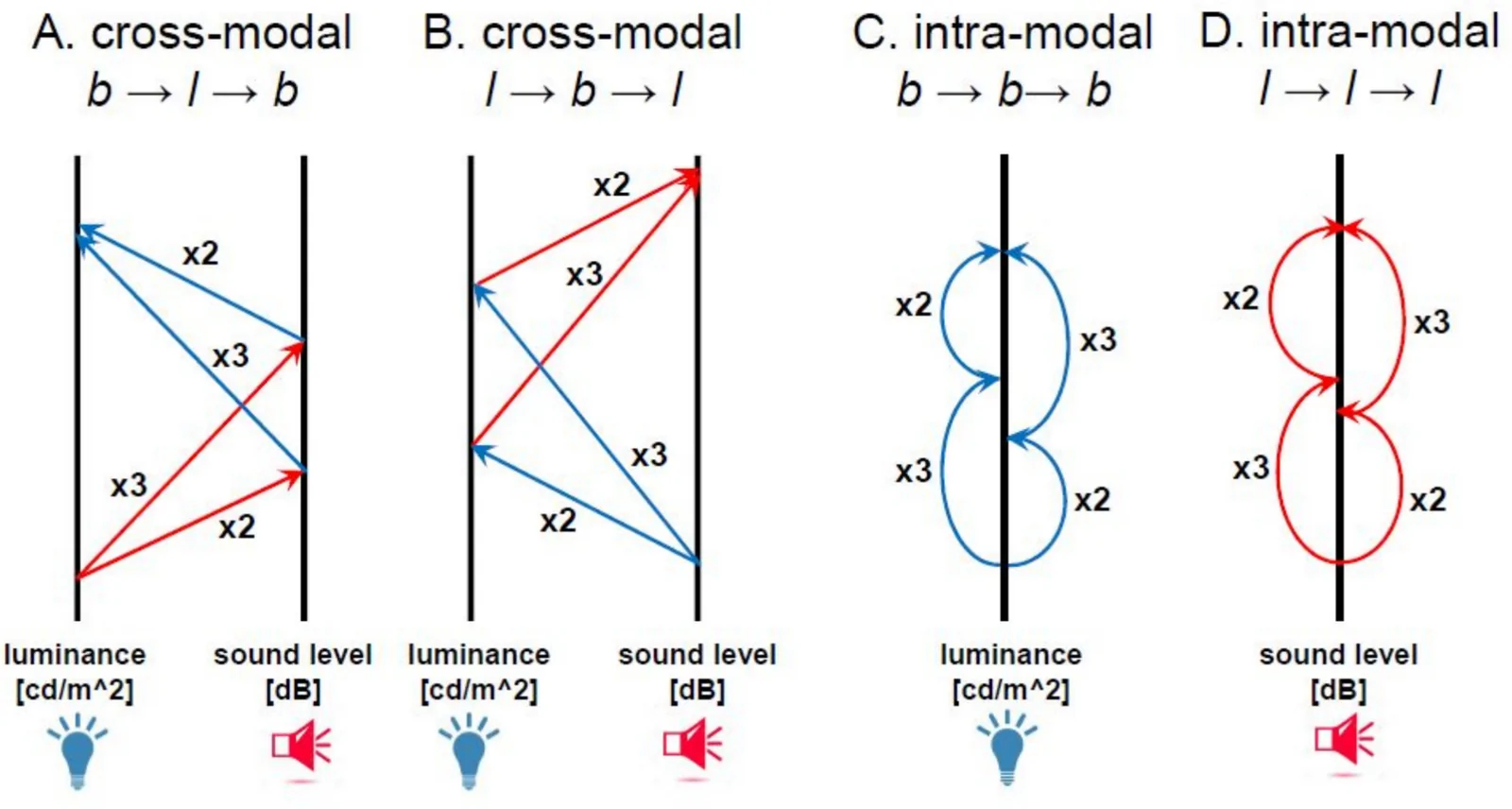
Four types of tests for commutativity of magnitude productions*.* The two graphs on the left depict adjustment sequences required for assessing cross-modal commutativity with the final outcome being (**A.**) a cross-modal brightness production (*x*^*blb*^) or (**B.**) a cross-modal loudness production (*x*^*lbl*^). The two sketches on the right illustrate adjustment sequences for determining intra-modal commutativity of (**C.**) brightness productions (*x*^*bbb*^) and (**D.**) loudness productions (*x*^*lll*^)

Ellermeier et al. (2021) did not perform another test of the convergence with intramodal adjustments suggested in Proposition 4 of Luce et al. (2010), that is: To compare cross-modal (Equations 2 and 3) with intra-modal (Eq. 1) adjustments involving the same magnitude production sequence (i.e., comparing adjustment sequence A with C and B with D, as illustrated in Fig. 1). Formally, that may be stated as evaluating the equivalence:

4 $${x}_{p,q}^{fgf} \sim {x}_{p,q}^{fff}.$$

Note that if participants used a single internal yardstick on which to map their sensation magnitudes and role-independent reference points on the two continua, these two sequences of magnitude productions $f\to g\to f$ and $f\to f\to f$ should coincide.

## Goals of the present study

The present study was thus designed

1. to replicate our earlier investigation of cross-modal commutativity of brightness and loudness productions with a new sample and improved statistical procedures,
2. to examine whether the net results of numerically identical cross-modal and intra-modal magnitude production sequences (e.g.: 2×3× adjustments) generated by the same participants do in fact converge, and
3. to interpret the pattern of outcomes in terms of the internal reference points observers are assumed to set when making cross-modal magnitude productions.

## Method

### Participants

Thirteen participants, including the first author, completed the experiment. The sample consisted of four women and nine men ranging in age between 18 and 65 years (*MD* = 23). Most of the participants were students of psychology or cognitive science who took part for course credit. All reported normal hearing and normal or corrected-to-normal vision. The protocol of the present research was submitted to the central ethics commission of the Technical University of Darmstadt and found to be “an uncritical psychological study” (TU Darmstadt EK 24/2019).

### Apparatus and stimuli

The experiment was conducted in a double-walled, sound-attenuated chamber (iac acoustics, Niederkrüchten, Germany) situated in a quiet laboratory room. Stimulus presentation and response registration were programmed in MATLAB utilizing the Psychophysics Toolbox extensions (Brainard, 1997; Pelli, 1997).

The sounds to be adjusted were digitally generated 500-ms bursts of pink noise with rise/decay times of 10 ms. They were D/A converted by an external sound card (RME Multiface II) with 16-bit resolution at a sampling rate of 44.1 kHz, passed through a headphone amplifier (Behringer HA 8000 Powerplay PRO-8) and played back diotically via electrodynamic headphones (Beyerdynamics DT 990 PRO). Sound levels were verified at the headphones using a sound level meter (Brüel & Kjær 2250) and an artificial ear (Brüel & Kjær Type 4153).

The light sources to be adjusted were luminous 5.7 x 5.7 cm grayscale squares presented on a regular TFT monitor (1,280 × 1,024 pixels) on a black background (approx. 0.2 cd/m^2^). The pixel intensity of the square was adjustable between 0 (black) and 255 (white), corresponding to luminance values of 0.2 cd/m^2^ and 85 cd/m^2^. Luminance levels were measured using a photometer (L 1009, Lichtmesstechnik Berlin) and resulted in a very good fit to a power function relating pixel intensity (*P*) to luminance (*L*_*v*_) by

5 $${L}_{v}=0.003\times {P}^{ 1.857}-1.076.$$

## Procedure

### Types of trials for testing commutativity

Evaluating commutativity both across and within modalities requires implementing different types of magnitude production trials, which are illustrated in Fig. 1. *Basic* trials are 2×, and 3× adjustments originating from standard levels of 40 dB(A) for pink noise and 1.66 cd/m^2^ for the luminance of the square (bottom set of arrows in each of the four graphs in Fig. 1) producing the respective sensation magnitude in the same (Graphs C and D in Fig. 1) or the other modality (Graphs A and B). *Successive* trials build upon a stimulus level produced on an earlier trial, which serves as the standard based on which the perceived magnitude of the comparison stimulus is adjusted to be 2× or 3× as intense (the top arrows in each of the four graphs in Fig. 1). These 16 types of trials (eight basic, eight successive; represented by all 16 arrows in Fig. 1) were randomly mixed in a block of trials, with the obvious constraint that a given successive trial be preceded by the basic trial it builds upon. Note that for *cross-modal* commutativity to hold, the final adjustments in Fig. 1A and 1B should be indistinguishable; whereas for *intra-modal* commutativity to hold that applies to graphs in Fig. 1C and 1D; while, of course, separately treating cases where the final adjustment is one of brightness (Fig. 1A and 1C), and where it is one of loudness (Fig. 1B and 1D).

### Structure of each adjustment trial

All trials (unlike in Ellermeier et al., 2021) used *successive* presentations of standard and comparison stimuli, in order to maintain the same trial structure for cross-modal and intra-modal adjustments.

On cross-modal trials (Fig. 1A and 1B), participants were asked to make a magnitude production either from brightness to loudness, or from loudness to brightness. To that effect, on a cross-modal loudness production trial, the digit “1” was presented on the screen for 300 ms—to indicate the first observation interval—followed by the display of the (standard) grayscale square for 500 ms. After a 500-ms interstimulus interval (ISI), the digit “2” appeared, followed by the 500-ms presentation of the (comparison) noise burst accompanied by the display of a loudspeaker symbol on the screen and an instruction (e.g., “Adjust the loudness of the sound to appear twice as intense as the brightness of the square!”). Subsequently, participants had unlimited time for making their response, increasing or decreasing the level of the comparison stimulus to be adjusted. For cross-modal magnitude productions of brightness, the same kind of sequence was used, only the order of visual and auditory stimuli was reversed.

Starting levels for the variable stimuli were randomly selected from the midrange—that is, between 50 and 65 dB(A) for sounds and between 0.5 and 50 cd/m^2^ for the luminous squares. Participants then adjusted the level of the variable stimulus by using two sets of “buttons” on the screen interface: Clicking the computer mouse on buttons labelled “>” and “>>” increased sound pressure levels by 1 and 6 dB, respectively, and pixel intensities by 3 and 15 units (on the scale from 0 to 255), to provide both small and large step sizes. Buttons labelled “<” and “<<” decreased levels by the same amounts. After participants had clicked one of the buttons, the audiovisual stimulus combination was repeated at the adjusted level, and so forth, until they pressed the “enter” key to indicate the match was satisfactory. When participants hit the limit of the permissible stimulus range—that is, 90 dB SPL or 85 cd/m^2^, a message “maximal loudness (or brightness) reached” was displayed.

For intramodal magnitude productions (as depicted in Fig. 1C and 1D), the procedure was essentially the same, except that two sounds (or luminous squares) were presented successively, with identical stimulus durations and timing, an ISI of 500 ms, and a pause of 500 ms before presentation of the next standard-comparison pair in the adjustment sequence.

### Session structure

Each participant completed the experiment in three sessions lasting approximately 45 to 60 min. In each session participants completed five blocks of trials—with optional pauses in-between—each of which contained the 16 trial types once, thus resulting in a total of 240 (3 × 5 × 16) magnitude production trials per participant. This way, fifteen ×2×3 sequences were accumulated to be compared with fifteen ×3×2 sequences for each of the four tests of commutativity per participant (as depicted in Fig. 1).

## Results

### A single subject’s data

**Fig. 2:**
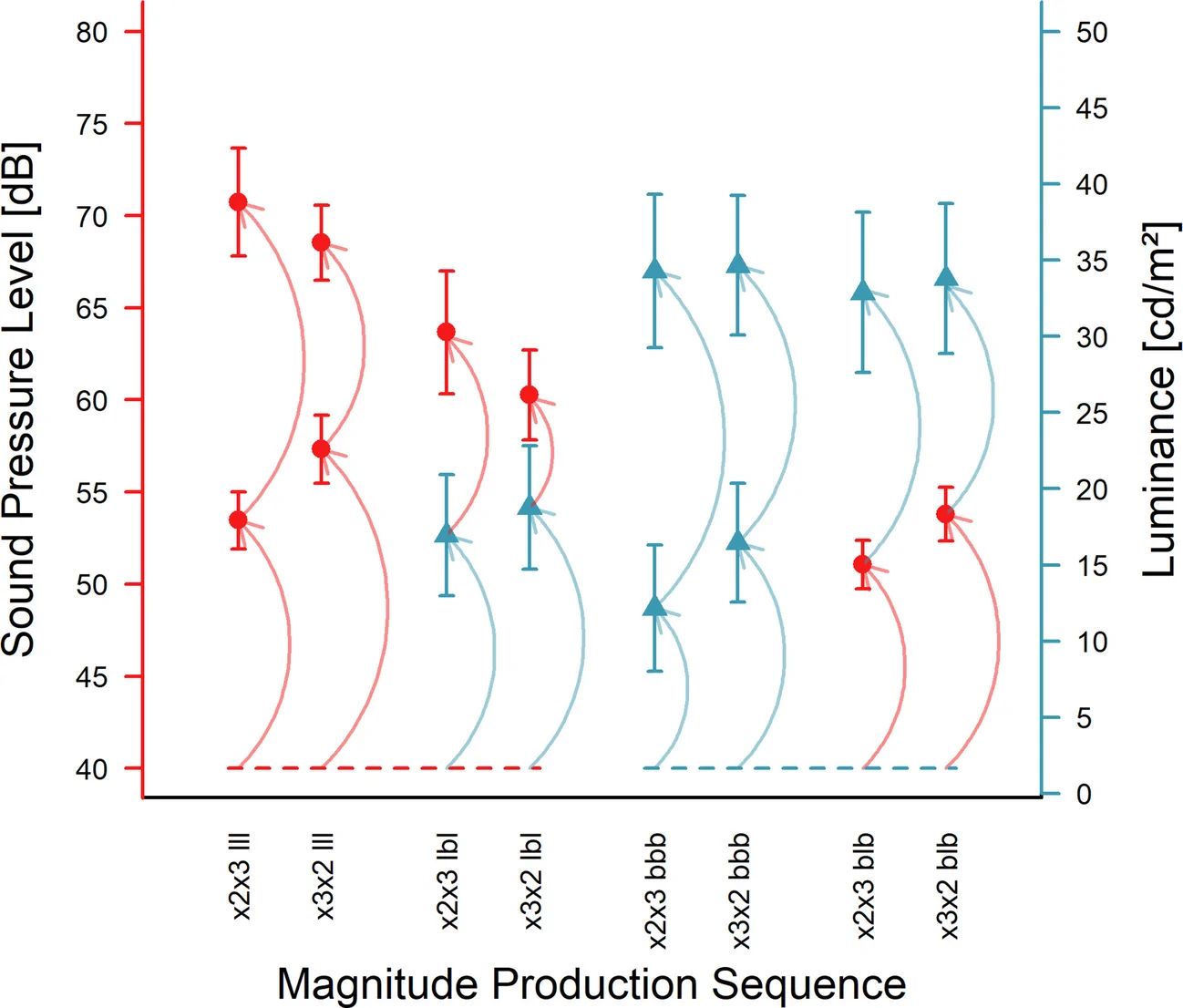
Magnitude productions produced by a single participant. Mean magnitude productions made by participant CORA08. The graph shows the mean adjustments made in the eight different sequences depicted in Fig. 1: Magnitude productions of loudness are represented by red arrows and refer to the left ordinate; magnitude productions of brightness are represented by blue arrows and refer to the right ordinate. Each arrow points to the arithmetic mean of 15 magnitude production trials and is plotted along with the respective 95% confidence interval

Each participant’s data were analyzed individually and all tests for commutativity were performed within subjects. Figure 2 shows an example of a single participant’s mean adjustments made in all of the 16 trial types sketched in Figure 1.

Inspecting Fig. 2, it is evident that the magnitude productions are relatively precise, their means (lower set of arrows) being ordered consistently with numerical instructions (*x*_*3*_ > *x*_*2*_), and nonoverlapping confidence intervals for successive adjustments. That is representative of the entire sample, in that across all participants, the average standard error of the mean (*SEM*) for the 15 adjustments per participant made in a given condition was as small as 3.58 cd/m^2^ for brightness productions and 1.89 dB for loudness productions.

For this particular participant, when the final adjustments were made on the brightness continuum (right ordinate in Fig. 2; upper set of arrows ending in blue triangles), the convergence of the successive adjustments shows that both cross-modal commutativity (comparing the two *blb* conditions) and intra-modal commutativity (the two *bbb* conditions) appear to hold. By contrast, when the final adjustments are magnitude productions of loudness (pointing to the left ordinate in Fig. 2; upper set of red circles), the adjustments fail to show clear evidence of commutativity, in that consecutive ×2×3 and ×3×2 adjustment sequences produce somewhat discrepant net results (for statistical evaluation of these differences, see Tables 1, 2, 3, 4). Furthermore, while the final adjustments of the intra-modal and cross-modal adjustment sequences appear to converge when the final target continuum is brightness (right ordinate), they are discrepant when the final target continuum is loudness, with the endpoints of intra-modal adjustments exceeding those of the cross-modal adjustments by more than 5 decibels (left ordinate). This equivocal outcome for a single participant strongly suggests to analyze each data set individually before pooling data.

**Table 1:** Individual tests for cross-modal commutativity

	Sequences ending in luminance adjustments [cd/m^2^]				Sequences ending in adjustments of SPL [dB]			
Participant	*×2×3 blb*	*×3×2 blb*	*p*	BF_01_	*×2×3 lbl*	*×3×2 lbl*	*p*	BF_01_
CAMA15	23.65 (8.39)	29.84 (9.99)	**.09**	0.87	65.53 (4.81)	63.33 (6.86)	.14	1.27
CORA08	32.87 (10.39)	33.79 (9.71)	.93	*3.70*	63.67 (6.58)	60.27 (4.82)	**.06**	0.46
DAPE12	21.53 (7.41)	28.79 (14.32)	.26	1.22	56.67 (3.44)	55.8 (4)	.64	3.11
EDRA17	47.23 (23.16)	47.84 (17.77)	.93	*3.78*	62.73 (8.56)	61.2 (6.07)	.23	1.92
EVTH19	39.41 (7.23)	32.16 (10.1)	**.03**	0.54	78.33 (7.22)	77.07 (6.87)	.55	2.88
INMA12	30.84 (11.49)	24.59 (7.15)	**.06**	0.56	58.93 (3.31)	57 (2.62)	**.09**	0.92
IRIS08	22.96 (13.68)	23.44 (9.33)	1.00	*3.79*	63 (5.44)	60.07 (6.15)	**.07**	0.69
JEER12	29.21 (10.63)	33.14 (14.92)	.24	2.58	78.33 (6.28)	75 (8.95)	.27	1.42
MORE12	25.18 (8.34)	24.29 (8.51)	1.00	*3.67*	57.47 (4.93)	56.4 (4.64)	.23	1.68
PEBE28	35.54 (11.12)	35.48 (9.64)	1.00	*3.81*	66.8 (8.97)	58.27 (6.03)	**.00**	**0.04**
RUTI08	45.92 (12.26)	41.69 (16.57)	.16	2.46	74.4 (6.02)	72.67 (5.67)	.22	1.64
SITO23	62.04 (19.21)	61.46 (21.52)	.94	*3.79*	70.67 (3.85)	67.07 (4.3)	**.01**	**0.03**
INHO28	42.74 (6.24)	38.63 (13.54)	.41	2.51	63.93 (3.08)	62.07 (1.98)	**.02**	**0.18**

Mean final adjustments in two complementary commutative, cross-modal adjustment sequences (*×2×3* vs. *×3×2*) along with standard deviations (in parentheses). Left half of the table: Sequences ending in adjusting brightness (*blb*); right half of the table: Sequences ending in adjusting loudness (*lbl*); *p* values (*p* < .1 printed in boldface) are the result of Wilcoxon signed-rank tests for the *×2×3* adjustment distributions being different from the *×3×2* distributions. Bayes factors (BF_01_) indicate how likely the null hypothesis of commutativity to hold is, compared with the alternative hypothesis of its violation. BF_01_ < .32 are highlighted by boldface print; BF_01_ > 3.2 indicating commutativity to hold are printed in italics.

**Table 2:** Individual tests for intra-modal commutativity

	Sequences ending in luminance adjustments [cd/m^2]^				Sequences ending in adjustments of SPL [dB]			
Participant	*×2×3 bbb*	*×3×2 bbb*	*p*	BF_01	*×2×3 lll*	*×3×2 lll*	*p*	BF_01
CAMA15	31.3 (12.87)	30.62 (7.42)	.98	*3.67*	69.33 (13.1)	71.2 (9.61)	.95	2.91
CORA08	34.28 (9.96)	34.64 (9.04)	.80	*3.77*	70.73 (5.78)	68.53 (4.03)	**.02**	**0.21**
DAPE12	19.42 (5.11)	17.2 (5.64)	.27	1.97	61.33 (3.99)	60.2 (2.76)	.31	2.15
EDRA17	49.8 (20.19)	48.62 (15.45)	.75	*3.66*	72 (9.11)	70.8 (10.69)	.22	2.40
EVTH19	30.37 (6.2)	31.59 (8.01)	.57	*3.21*	83.8 (7.09)	83.4 (5.33)	.71	*3.61*
INMA12	33.21 (10.6)	36.21 (10.54)	.48	2.86	60.8 (3.86)	61.6 (3.68)	.53	3.17
IRIS08	33.59 (10.45)	35.96 (13.12)	.62	3.09	66.47 (7.21)	69.2 (7.78)	.90	2.69
JEER12	36.24 (15.85)	40.94 (10.95)	.32	2.48	77.53 (6.13)	75.27 (5.04)	.16	1.35
MORE12	27.06 (7.34)	26.24 (8.25)	.66	*3.40*	57.27 (4.62)	57.13 (4.42)	.85	*3.74*
PEBE28	44.7 (13.33)	47.35 (12.52)	.55	*3.31*	68.07 (5.54)	66.33 (3.46)	.30	1.75
RUTI08	62.45 (14.16)	63.74 (13.84)	.75	*3.70*	80.47 (6.42)	79.47 (5.34)	.19	1.31
SITO23	65.18 (16)	76.43 (11.93)	**< .01**	**0.01**	74.67 (4.7)	72.4 (9.03)	.33	1.99
INHO28	38.6 (12.14)	36.6 (8.64)	.41	2.64	65 (3.18)	64.87 (3.4)	.80	*3.68*

Mean final adjustments in two complementary commutative adjustment sequences (*×2×3* vs. *×3×2*) of brightness (*bbb*, left) and loudness (*lll*, right), along with standard deviations (in parentheses); *p* values (*p* < .1 printed in boldface) are the result of Wilcoxon signed-rank tests for the *×2×3* adjustment distributions being different from the *×3×2* distributions. Bayes factors (BF_01_) indicate how likely the null hypothesis of commutativity to hold is, compared with the alternative hypothesis of its violation. BF_01_ < .32 are highlighted in boldface; BF_01_ > 3.2 indicating commutativity to hold are printed in italics.

**Table 3:** Comparing cross-modal and intra-modal production sequences ending on sound pressure level

	×3×2 adjustments				×2×3 adjustments			
Participant	*3×2 lbl*	*3×2 lll*	*p*	BF_01	*2×3 lbl*	*2×3 lll*	*p*	BF_01
CAMA15	63.33 (6.86)	71.2 (9.61)	**< .01**	**0.01**	65.53 (4.81)	69.33 (13.1)	**.08**	1.70
CORA08	60.27 (4.82)	68.53 (4.03)	**< .01**	**< 0.01**	63.67 (6.58)	70.73 (5.78)	**.01**	**0.04**
DAPE12	55.8 (4)	60.2 (2.76)	**.01**	**0.07**	56.67 (3.44)	61.33 (3.99)	**.01**	**0.10**
EDRA17	61.2 (6.07)	70.8 (10.69)	**< .01**	**0.02**	62.73 (8.56)	72 (9.11)	**< .01**	**< 0.01**
EVTH19	77.07 (6.87)	83.4 (5.33)	**< .01**	**< 0.01**	78.33 (7.22)	83.8 (7.09)	**< .01**	**0.03**
INMA12	57 (2.62)	61.6 (3.68)	**< .01**	**0.01**	58.93 (3.31)	60.8 (3.86)	.14	1.17
IRIS08	60.07 (6.15)	69.2 (7.78)	**.01**	**0.07**	63 (5.44)	66.47 (7.21)	.12	1.75
JEER12	75 (8.95)	75.27 (5.04)	.97	*3.78*	78.33 (6.28)	77.53 (6.13)	.86	*3.51*
MORE12	56.4 (4.64)	57.13 (4.42)	.42	2.61	57.47 (4.93)	57.27 (4.62)	.75	*3.73*
PEBE28	58.27 (6.03)	66.33 (3.46)	**< .01**	**0.01**	66.8 (8.97)	68.07 (5.54)	.36	*3.41*
RUTI08	72.67 (5.67)	79.47 (5.34)	**< .01**	**0.01**	74.4 (6.02)	80.47 (6.42)	**< .01**	**< 0.01**
SITO23	67.07 (4.3)	72.4 (9.03)	**.01**	**0.14**	70.67 (3.85)	74.67 (4.7)	**.01**	**0.05**
INHO28	62.07 (1.98)	64.87 (3.4)	**< .01**	**0.03**	63.93 (3.08)	65 (3.18)	.10	1.36

Mean final SPLs of a cross-modal (*lbl*) and an intra-modal (*lll*) adjustment sequence of type “3×2” (left) or “×2×3” (right), along with their standard deviations (in parentheses); *p*-values (*p* < .1 printed in boldface) are the result of Wilcoxon signed-rank tests for the cross-modal adjustment distributions being different from the intra-modal ones. Bayes factors (BF_01_) indicate how likely the null hypothesis of the cross-modal and intra-modal adjustment sequences converging on similar SPLs is. BF_01_ < .32 are highlighted in boldface; BF_01_ > 3.2 indicating commutativity to hold are printed in italics.

**Table 4:** Comparing cross-modal and intra-modal production sequences ending on luminance

	×3×2 adjustments				×2×3 adjustments			
Participant	*blb 3×2*	*bbb 3×2*	*p*	BF_01	*blb 2×3*	*bbb 2×3*	*p*	BF_01
CAMA15	29.84 (9.99)	30.62 (7.42)	.89	*3.70*	23.65 (8.39)	31.3 (12.87)	**.05**	0.41
CORA08	33.79 (9.71)	34.64 (9.04)	.63	*3.67*	32.87 (10.39)	34.28 (9.96)	.85	*3.44*
DAPE12	28.79 (14.32)	17.2 (5.64)	**.02**	**0.20**	21.53 (7.41)	19.42 (5.11)	.29	1.80
EDRA17	47.84 (17.77)	48.62 (15.45)	.80	*3.74*	47.23 (23.16)	49.8 (20.19)	.78	3.17
EVTH19	32.16 (10.1)	31.59 (8.01)	.89	*3.75*	39.41 (7.23)	30.37 (6.2)	**.01**	**0.02**
INMA12	24.59 (7.15)	36.21 (10.54)	**< .01**	**0.02**	30.84 (11.49)	33.21 (10.6)	.38	*3.22*
IRIS08	23.44 (9.33)	35.96 (13.12)	**.02**	**0.16**	22.96 (13.68)	33.59 (10.45)	**.04**	0.62
JEER12	33.14 (14.92)	40.94 (10.95)	.13	1.23	29.21 (10.63)	36.24 (15.85)	.27	1.79
MORE12	24.29 (8.51)	26.24 (8.25)	.53	*3.30*	25.18 (8.34)	27.06 (7.34)	.36	3.13
PEBE28	35.48 (9.64)	47.35 (12.52)	**< .01**	**0.23**	35.54 (11.12)	44.7 (13.33)	.15	1.12
RUTI08	41.69 (16.57)	63.74 (13.84)	**< .01**	**0.01**	45.92 (12.26)	62.45 (14.16)	**< .01**	**0.01**
SITO23	61.46 (21.52)	76.43 (11.93)	**< .01**	**0.08**	62.04 (19.21)	65.18 (16)	.29	2.41
INHO28	38.63 (13.54)	36.6 (8.64)	.66	*3.25*	42.74 (6.24)	38.6 (12.14)	.22	1.85

Mean final luminances of cross-modal (*blb*) and intra-modal (*bbb*) adjustment sequences of type “×3×2” (left) or “×2×3” (right), along with their standard deviations (in parentheses); *p*-values (*p* < .1 printed in boldface) are the result of Wilcoxon signed-rank tests for the cross-modal adjustment distributions being different from the intra-modal ones. Bayes actors (BF_01_) indicate how likely the null hypothesis of the cross-modal and intra-modal adjustment sequences converging on similar luminance levels is. BF_01_ < .32 are highlighted in boldface; BF_01_ > 3.2 indicating commutativity to hold are printed in italics.

### Statistical analysis

In order to statistically analyze the different kinds of cross-modal and intra-modal commutativity, two strategies were employed: (1) nonparametric null hypothesis testing for deviations from commutativity, and (2) Bayesian statistics to estimate the likelihood of commutativity to hold. These statistical analyses were performed on the individual sound-pressure level adjustments in decibels and on the grayscale adjustments after converting the recorded pixel intensity to luminance in candela per m.^2^

Commutativity was evaluated using Wilcoxon signed-rank tests to compare each individual’s 3×2× successive adjustments with their 2×3× adjustments. Matched-pairs nonparametric tests were employed rather than their counterparts for independent samples, to better account for potential drifts in the adjustments in the course of the experiment.

Since the commutativity axiom (Equations 2 and 3) basically claims that the net result of two different consecutive adjustments (×2×3 and ×3×2) should match, which amounts to attempting to show that the null hypothesis holds, a supplementary strategy better suited to assess the likelihood of the null appeared necessary. To that effect, Bayes factors (BF_01_) were computed for each participant’s data using the *BayesFactor* package for R (Morey & Rouder, 2011, 2024; Morey et al., 2011; Rouder et al., 2012) in order to determine the likelihood of commutativity to hold (i.e., the null hypothesis; Model 0) relative to an axiom violation (the alternative hypothesis; Model 1). All Bayes factors were determined with the “ttestBF()” function for paired observations using wide, and thus relatively uninformed Cauchy prior distributions around a standardized effect size of 0 (width scaled with γ = √2/2; as suggested by Morey et al., 2011; Rouder et al., 2012). Thus, as applied to the present analysis, Bayes factors (BF_01_) exceeding 1.0 in principle favor the null hypothesis implying the equality of two sequences of magnitude productions, or commutativity to hold. By convention, however, Bayes factors 0.32 < BF_01_ < 3.16 are not considered evidence worth mentioning for either hypothesis (e.g., Jeffreys, 1961/1998, p. 432).

### Mean magnitude productions

**Fig. 3:**
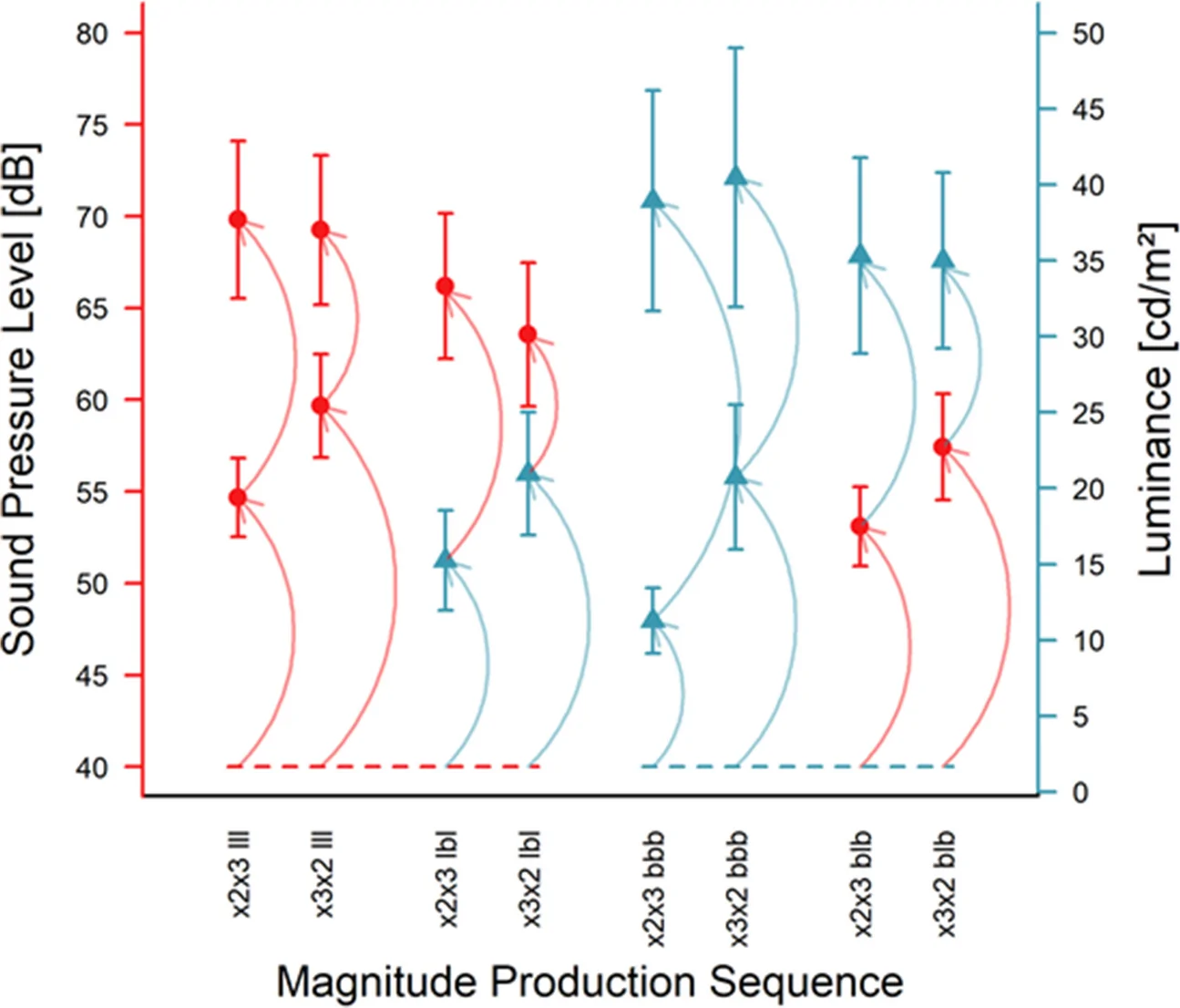
Mean cross-modal and intra-modal magnitude productions. Overall means and 95%-confidence intervals of the magnitude productions made by all *N* = 13 participants in the 16 experimental conditions. The labels on the abscissa denote the magnitude production sequence in question, with, for example, “×2×3 lbl” referring to first doubling, then tripling perceived magnitude, and proceeding from loudness (l) to brightness (b) and back to loudness (l). Red arrows ending in filled circles denote magnitude productions of loudness (referring to the left ordinate) and blue arrows ending in filled triangles denote magnitude productions of brightness (referring to the right ordinate). The initial magnitude productions originated from a reference sound pressure level of 40 dB(A) and a reference luminance level of 1.66 cd/m^2^ (dashed lines). Each data point is based on 13 × 15 = 195 adjustments

In order to visualize the overall empirical outcome, the average adjustments made by all 13 participants in each of the 16 conditions are depicted in Figure 3, illustrating the descriptive outcome for the different kinds of commutativity considered.

In line with the monotonicity assumption (see Ellermeier et al., 2021), the average adjustments of both sound pressure level and luminance increased from ×2 to ×3 productions in the cross-modal (*M*_*x2*_ = 53.1 to *M*_*x3*_ = 57.4 dB SPL and *M*_*x2*_ = 15.2 to *M*_*x3*_ = 21.4 cd/m2) and in the intra-modal conditions (*M*_*x2*_ = 54.4 to *M*_*x3*_ = 59.7 dB SPL and *M*_*x2*_ = 11.3 to *M*_*x3*_ = 20.7 cd/m2). On an individual level, the mean adjustments were in line with monotonicity for all 13 participants in the intra-modal conditions as well as for the cross-modal adjustments of sound pressure level, whereas there was a violation of monotonicity in only one participant for cross-modal luminance adjustments (i.e., 98% of the individual mean adjustments increased monotonically from ×2 to ×3 productions).

Whether, on average, cross-modal commutativity tends to hold, may be inferred from Fig. 3 by comparing the net result of making an ×2×3 vs. an ×3×2 adjustment (cf. the endpoints of the adjustment sequences containing “mixed” symbols/colors). While, for luminance being the final target continuum (the two *blb* sequences in Fig. 3), mean numerical values of the composite adjustments involving commutativity almost coincide (*M*_*×2×3*_ = 35.2 vs. *M*_*×3×2*_ = 35.0 cd/m^2^), they are somewhat discrepant for sound pressure level constituting the target continuum (the two *lbl* sequences: *M*_*×2×3*_ = 66.2 vs. *M*_*×3×2*_ = 63.6 dB SPL).

Intra-modal commutativity should hold, on average, if the two *lll* (and the two *bbb*) sequences, respectively, converge on roughly the same stimulus levels, which, given statistical error, appears to be the case for both luminance and sound pressure level (see Fig. 3).

### Statistical evaluation of cross-modal and intra-modal commutativity

To statistically decide whether both kinds of commutativity are valid for each individual subject and for starting out with either modality, Wilcoxon signed-rank tests were performed with a significance level of α = 0.1.

As may be seen in Table 1, based on the nonparametric tests, cross-modal commutativity was violated in 9 of 26 (13 participants × 2 target modalities) tests. The Bayesian statistics are equally inconclusive overall: While 17 of the 26 tests suggest that the null hypothesis of commutativity to hold is more likely than an axiom violation (BF_01_ > 1), in only 6 tests of cross-modal commutativity is the null hypothesis found to be more than three times as likely as the alternative hypothesis (BF_01_ > 3.2; see Table 1).

Intra-modal commutativity may be inspected in Table 2: Here, just two of 26 nonparametric tests indicate a significant axiom violation. Based on the Bayesian statistics, the null hypothesis is more than three times more likely than the alternative hypothesis (an axiom violation) in 10 of the 26 tests.

## Comparing cross-modal with intra-modal magnitude productions

Inspection of Fig. 3 suggests that the (largely commutative) cross-modal and intra-modal adjustment sequences do not converge at the same absolute luminance (right ordinate) or sound pressure (left ordinate) levels, as is postulated by Equation 4. Rather, for both target dimensions, mean intra-modal (same plot symbol and color) adjustment sequences appear to substantially overshoot the corresponding cross-modal sequences (changing symbols and colors). To check for the significance of this discrepancy in each individual case—in accordance with Equation 2—four additional sets of comparisons were performed, depending on whether the adjustment sequence was ×2×3 or ×3×2, and whether the final target dimension was sound (Table 3) or light (Table 4).

When the final adjustments were of sound pressure levels (Table 3), 18 of the 26 tests (*×2×3* or *×3×2* for 13 participants each) indicated significant discrepancies between cross-modal and intra-modal sequences. By the same token, only four of 26 Bayes factors exceeded 3.2, favoring the null hypothesis. When the final adjustments were made in luminance (Table 4), 10 of the 26 tests showed significant differences between the net result of intra-modal vs. cross-modal sequences, and 8 of 26 Bayesian tests favored the null hypothesis over the alternative by a factor greater than 3.2. Note that in the data presented in Tables 3 and 4, the statistical conclusions to be drawn from the “frequentist” statistics and the Bayesian analysis remarkably agree in suggesting a lack of convergence of intra-modal and cross-modal production sequences.

## Discussion

The results of the present axiomatic investigation of cross-modal commutativity of magnitude productions extend our earlier study (Ellermeier et al., 2021) by (1) replicating cross-modal commutativity with a new sample, (2) obtaining data on intra-modal commutativity from those same participants, and (3) comparing concatenations of cross-modal versus intra-modal magnitude productions, the final outcome of which, according to the Luce et al. (2010) theory, should coincide (Equation 4), if participants indeed rely on the same internal references when making cross-modal and intra-modal magnitude productions.

## Cross-modal commutativity

Based on nonparametric null-hypothesis testing, commutativity of cross-modal magnitude productions (Equations 2 and 3) was found to be violated in nine out of 26 tests (35%) which is somewhat higher than the proportion of axiom violations (17.5%) that had been found by Ellermeier et al. (2021). A parallel Bayesian analysis of the present data taking the critique by Heller (2025) into account by using updated parameters based on Rouder et al. (2012) found merely three of the 26 tests to favor the alternative hypothesis (BF_01_ < 0.32). Substantial evidence, however, favoring the null hypothesis (of cross-modal commutativity to hold) was only found in six of these tests (BF_01_ > 3.2). Thus, the evidence regarding the present Research Goal 1 (cross-modal commutativity) remains somewhat inconclusive.

The relatively small number of axiom violations (and the considerable number of inconclusive Bayes factors) may be taken as indicating insufficient statistical power (see Heller, 2025), but note that a close replication of Ellermeier et al. (2021) with increased power due to a larger number of observations (Kohler, 2024; Experiment 1; Kohler, personal communication, October 9, 2025) found proportions of commutativity violations (31%) coming very close to those seen in the present data collection.

**Fig. 4:**
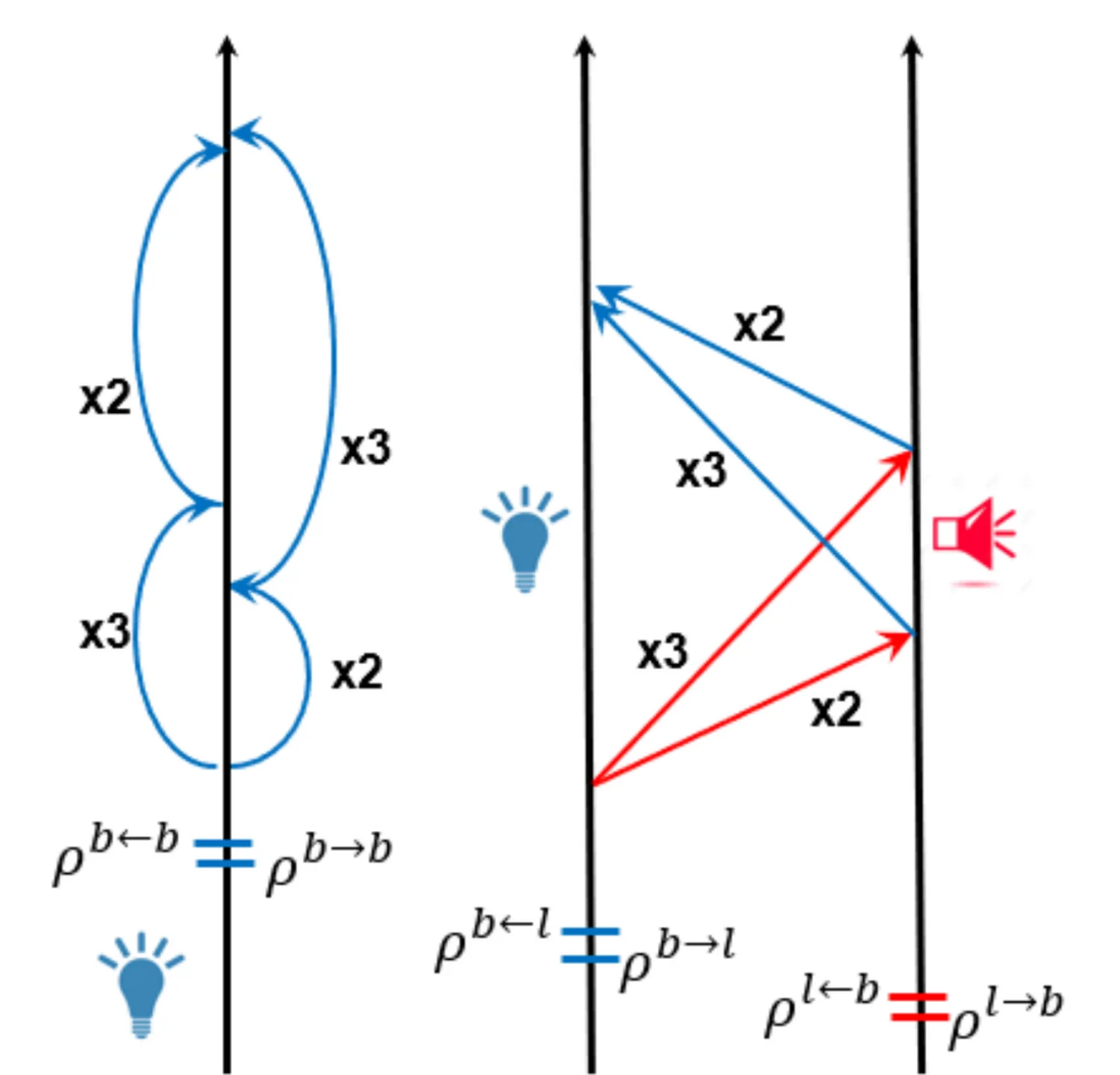
Hypothetical representation of the reference points involved. The figure depicts how internal “reference points” might come into play when making intra-modal (left) and cross-modal (right) magnitude productions. It is drawn so that the reference points involved in intra-modal (leftmost axis) or cross-modal magnitude productions (center and rightmost axis) are role-independent and thus relatively close to each other, resulting in commutative magnitude production sequences both within and across modalities. By contrast, the reference points on the brightness continuum for intra-modal (left) and cross-modal (center) productions are further apart, producing the disparities seen in the actual data (Fig. 3).

The nonnegligeable number of axiom violations with respect to cross-modal commutativity and the relatively small number of cases where commutativity appears highly likely based on the Bayesian analysis (see Table 1) requires an explanation. The limited theoretical literature suggests to base it on the concept of “internal reference points” originally introduced by Luce et al. (2010, Proposition 4), and recently extended by Heller (2021). These reference points might not exhibit “cross-dimensional role independence” (Heller, 2021, Proposition 2) for all participants, in that the internal reference established on the loudness continuum ${\rho }^{l\leftarrow b}$ when starting from a given brightness and making the comparison sound “three times as loud as the light is bright”, might not be the same as when proceeding the other way, from loudness to brightness, ${\rho }^{l\to b}$. Only if these reference points agree (${\rho }^{l\to b}={\rho }^{l\leftarrow b}$)—that is, are independent of whether *l* is the standard or (variable) comparison—will commutativity result. Note that when Heller (2025) reanalyzed our earlier data on cross-modal commutativity (Ellermeier et al., 2021) collected under identical conditions as in the present experiment, he estimated model parameters suggesting that observers base their adjustments on systematically higher reference intensities in the variable or “target” modality than in the “standard” modality from which the magnitude productions originate, as is schematically illustrated in Fig. 4. Such a systematic offset in internal reference points might tentatively account for the axiom violations seen in cross-modal commutativity.

### Intra-modal commutativity

Evaluating commutativity of magnitude productions separately for brightness and loudness found it to hold in the majority of tests as had previous investigations of intra-modal commutativity for these two modalities (loudness: Ellermeier & Faulhammer, 2000; Steingrimsson & Luce, 2005, 2007; Zimmer, 2005; brightness: Peißner, 1999; Steingrimsson, 2009, 2011; Steingrimsson et al., 2012). Note that one of the two significant violations of intra-modal commutativity occurring in 26 tests is seen in Fig. 2, where the ×2×3-lll sequence exceeds the ×3×2-lll sequence on average by approximately 3 decibels.

### Comparing cross-modal with intramodal production sequences

The results of the present investigation show that on average, intra-modal ×2×3 (or ×3×2) adjustment sequences do not converge with the corresponding cross-modal adjustment sequences; as is seen in Fig. 3 (compare the cross-modal, red-and-blue sequences with the unimodal, monochromatic sequences), the former exceed the latter systematically, on average by some 3.62 decibels (or 5.71 dB for the ×3×2 adjustments) when sound is the final target dimension. Only 9 of 52 comparisons (13 participants × 2 final target modalities × 2 sequences) constitute exceptions to this effect, in that cross-modal sequences exceed the mean adjustments made in intra-modal sequences (see Tables 3 and 4). This pattern of outcomes provides a clear answer with respect to Research Goal 2, in that intramodal and cross-modal adjustment sequences do not appear to converge.

Though this critical comparison, stated formally in Equation 4, has not been performed with truly cross-modal magnitude productions before (i.e., involving different sensory modalities such as vision and audition), the two landmark studies in this area reported similar results for what they called “cross-dimensional” magnitude productions involving the loudness of tones of different frequencies (e.g., “Make the high-pitched tone twice as loud as the low-pitched tone”; Luce et al., 2010) or the luminance of different hues (Steingrimsson et al., 2012). In both studies, cross-dimensional production sequences (left side of Equation 4) disagreed with unidimensional magnitude productions (right side of Equation 4) for the relatively small number of participants tested.

### The role of internal reference points

The discrepancies between intramodal and cross-modal magnitude production sequences might also be attributed to the task dependence of the internal reference points (or “reference signals”; Luce et al., 2010) adopted. In terms of Heller’s (2021) theory, its Proposition 3 requires that for ${x}_{p,q}^{blb} \sim {x}_{p,q}^{bbb}$ to hold, not only are cross-dimensional role independence (the left side of Equation 5, Heller’s Proposition 2) and intra-dimensional role independence required (the right side of Equation 5, Heller’s Proposition 1), but also *cross-dimensional s-invariance*, ${\rho }^{b\to l}={\rho }^{b\to b},$ and *cross-dimensional v-invariance*, ${\rho }^{b\leftarrow l}={\rho }^{b\leftarrow b}$ (Heller, 2021, Proposition 3), meaning that the reference points adopted when making a cross-modal magnitude production must be the same as when making an intramodal magnitude production, no matter if the continuum in question functions as the standard or the to-be-adjusted comparison. Thus, if the internal reference used in a cross-modal magnitude production does not coincide with the internal reference employed in an intra-modal production (i.e., ${\rho }^{b\to l}$≠ ${\rho }^{b\to b}$; as schematically illustrated in Fig. 4), the invariance postulated in Equation 5 will be violated, as is the case for most observers in the present study (Tables 3 and 4).

When comparing individual participants across the tests performed (i.e., taking advantage of the fact that all participated in each task in the present study), no particularly striking pattern emerges. There are two participants (JEER12 and MORE12) for whom no significant axiom violation occurs in any of the conditions tested (see Tables 1–4), and for whom the Bayes factors also favor the null in most instances. Furthermore, five participants (DAPE12, EDRA17, JEER12, MORE12, and RUTI08) satisfy both cross-modal (Table 1) and intramodal commutativity (Table 2) by frequentist significance tests, but (except for JEER12 and MORE12) show significant discrepancies between cross-modal and intramodal adjustment sequences (Tables 3 and 4). This is consistent with the idea that the lack of convergence of the cross-modal and intramodal adjustments is *not* due to failures to comply with cross-modal or intramodal commutativity, but rather to the lacking *cross-dimensional s-invariance* and *cross-dimensional v-invariance* (Heller, 2021, Proposition 3), as discussed in the previous paragraph.

### Existence of a universal internal magnitude scale

What does this state of affairs leave us with to conclude? As Luce et al. (2010) formulate the question in their article that triggered the research on cross-modal magnitude production, “can loudness and brightness each be considered to be special cases of a common ratio scale of subjective magnitude for both modalities?” (p. 1249). While this idea is only implicit in Stevens’s belief in the universality of cross-modality matching (Stevens 1959, 1975, p. 100; Teghtsoonian, 1971, 2012; but see Spence & Di Stefano, 2024), it has been formally stated in Stevens’s reinterpretation in terms of axiomatic measurement theory (Luce et al., 2010; Narens, 1996; Steingrimsson et al., 2012). Interestingly, the introduction of (disparate) reference points makes failures to find commutativity commensurable with the idea of observers operating on a common underlying ratio scale. Note that according to Heller’s (2021, 2025) conceptualization, commutativity may only be found if the internal reference points used in making magnitude productions are equal, but violations of the equality of reference points (and ensuing violations of commutativity) do not preclude that participants are operating on a common ratio scale of perceived intensity when making magnitude productions from loudness to brightness and vice versa. More specifically, the present data analysis suggests that while both intra-modal and cross-modal magnitude productions are largely commutative, participants appear to use different internal references depending on whether they make magnitude productions within a given modality, say, loudness, as opposed to across modalities, say, from light to sound.

Of course, postulating disparate internal reference points (Heller, 2021, 2025; Luce et al., 2010) to explain the remaining violations of cross-modal commutativity, as well as the prevailing discrepancy between chaining cross-modal versus chaining intra-modal magnitude productions, is still a post-hoc account open to alternative explanations (e.g., by context effects). Backing up the present theoretical account might require a way to experimentally manipulate the choice of the internal reference intensities participants supposedly base their magnitude productions on, either by instruction, or by procedure, such as by providing explicit external references, for example, in the background illumination or the ambient noise level. Furthermore, different perceptual continua—other than loudness and brightness which share a number of properties (e.g., similar dynamic ranges or psychophysical power functions)—should be investigated, and a greater variety of “starting levels” for the successive ratio productions than in the present study should be explored.

## Data Availability

The individual data sets of all participants in the current study (all SPL and luminance adjustments made by each participant) are openly available in an OSF repository (https://osf.io/5avbw/?view_only=687aef266f174648863f86b4982e35b9), as are the condition means per participant. The study has not been preregistered.
